# Hydrogen peroxide-activatable antioxidant prodrug as a targeted therapeutic agent for ischemia-reperfusion injury

**DOI:** 10.1038/srep16592

**Published:** 2015-11-13

**Authors:** Dongwon Lee, Seunggyu Park, Soochan Bae, Dahee Jeong, Minhyung Park, Changsun Kang, Wooyoung Yoo, Mohammed A. Samad, Qingen Ke, Gilson Khang, Peter M. Kang

**Affiliations:** 1 Department of BIN Convergence Technology, Chonbuk National University, Jeonju, Chonbuk 561-756, Republic of Korea; 2 Polymer Fusion Research Center, Department of Polymer·Nano Science and Technology, Chonbuk National University, Jeonju, Chonbuk 561-756, Republic of Korea; 3 Cardiovascular Institute, Beth Israel Deaconess Medical Center and Harvard Medical School, Boston, MA 02215, United States

## Abstract

Overproduction of hydrogen peroxide (H_2_O_2_) causes oxidative stress and is the main culprit in the pathogenesis of ischemia/reperfusion (I/R) injury. Suppression of oxidative stress is therefore critical in the treatment of I/R injury. Here, we report H_2_O_2_-activatable antioxidant prodrug (BRAP) that is capable of specifically targeting the site of oxidative stress and exerting anti-inflammatory and anti-apoptotic activities. BRAP with a self-immolative boronic ester protecting group was designed to scavenge H_2_O_2_ and release HBA (*p*-hydroxybenzyl alcohol) with antioxidant and anti-inflammatory activities. BRAP exerted potent antioxidant and anti-inflammatory activity in lipopolysaccharide (LPS)- and H_2_O_2_-stimulated cells by suppressing the generation of ROS and pro-inflammatory cytokines. In mouse models of hepatic I/R and cardiac I/R, BRAP exerted potent antioxidant, anti-inflammatory and anti-apoptotic activities due to the synergistic effects of H_2_O_2_-scavenging boronic esters and therapeutic HBA. In addition, administration of high doses of BRAP daily for 7 days showed no renal or hepatic function abnormalities. Therefore BRAP has tremendous therapeutic potential as H_2_O_2_-activatable antioxidant prodrug for the treatment of I/R injuries.

Hydrogen peroxide (H_2_O_2_) is an essential oxygen metabolite and serves as a messenger in cellular signal pathways that are necessary for the growth, development and fitness of living organisms^1^,^2^. However, H_2_O_2_ is one of reactive oxygen species (ROS) and its aberrant accumulation causes oxidative stress and inflammation events, which are highly correlated with the onset and development of various pathological conditions such as cancer, diabetes, cardiovascular diseases and ischemia-reperfusion (I/R) injury^3^,^4^,^5^. I/R injuries are seen in a variety of clinical conditions such as acute coronary syndrome, hepatic and renal ischemic insults, cardiopulmonary bypass surgery and vascular thromboembolic events^6^,^7^,^8^,^9^. Reperfusion of blood flow into ischemic tissues induces a large generation of H_2_O_2_ which is one of the most common ROS and causes oxidative stress and cellular damages, further exacerbating tissue damages^10^,^11^. Thus, H_2_O_2_ is an attractive target of oxidative stress-associated diseases and targeted therapy directed to the site of I/R injury, which is characterized by high concentration of H_2_O_2_ will offer significant advantages over generalized antioxidant therapy.

*p*-Hydroxybenzyl alcohol (HBA) is one of major active components of *Gastrodia elata*, which is a widely used herbal agent for the treatment of inflammatory diseases and convulsive disorders in oriental countries^12^. HBA exerts antioxidant activities and plays a protective role against oxidative stress-related diseases such as coronary heart diseases and ischemic brain injury^13^,^14^. HBA is also a powerful scavenger of hydroxyl radical and superoxide due to its phenolic hydroxyl group^15^,^16^. Therefore, there has been a great interest in the use of HBA as an antioxidant and therapeutic agent^17^,^18^,^19^,^20^,^21^. However, HBA is unable to scavenge H_2_O_2_ and has a short blood circulation time, ~30 min of half-life, which would limit its clinical applications^22^.

Medical research has been consistently seeking to develop drugs which have temporal and spatial control of therapeutic activities and exhibit desired pharmacological effects^23^. Prodrugs are a class of bioreversible therapeutic molecules that undergo enzymatic or chemical transformation *in vivo* to generate the active drug, providing targeted therapeutic activities^24^,^25^. In this regard, we developed BRAP as a H_2_O_2_-activatable antioxidant prodrug of HBA, which can scavenge H_2_O_2_ and exert anti-inflammatory and anti-apoptotic activities. Herein, we report the potential of BRAP as an I/R targeted therapeutic agent using a cell culture model and animal models of I/R injuries.

## Results

### Synthesis and characterization of BRAP

**Figure 1:**
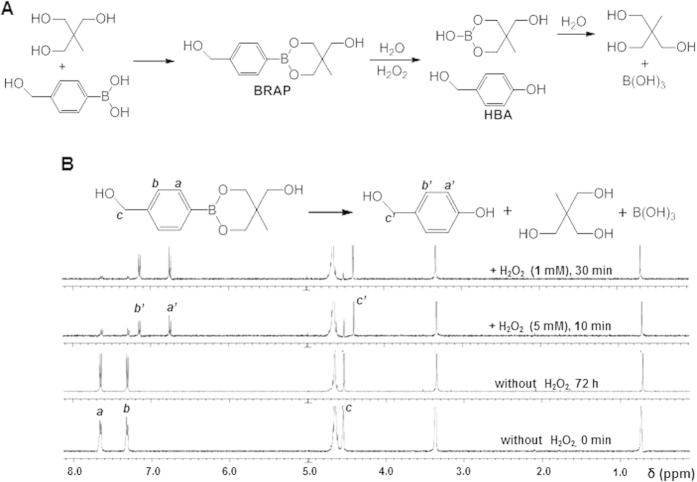
Generation of H_2_O_2_-scavenging antioxidant BRAP. (**A**) A synthetic route and degradation of BRAP as a H_2_O_2_-activatable antioxidant prodrug. (**B**) ^1^H NMR spectra of BRAP before and after H_2_O_2_-mediated hydrolysis.

H_2_O_2_-activatable antioxidant prodrug BRAP was synthesized from a simple reaction of (4-(hydroxymethyl)phenyl)boronic acid and 2-(hydroxymethyl)-2-methylpropane-1,3-diol at room temperature (Fig. 1A). BRAP was obtained as fine white powder and its chemical structure was confirmed by NMR (Fig. 1B; Supplementary Fig. S1). Since BRAP containing boronic ester was designed to be readily oxidized by H_2_O_2_ to generate HBA, boric acid and 2-(hydroxymethyl)-2-methylpropane-1,3-diol, we investigated the sensitivity of BRAP to H_2_O_2_ using ^1^H NMR. BRAP was added to D_2_O containing H_2_O_2_ and the changes in the signal were monitored over time. In the presence of H_2_O_2_, BRAP was oxidized to generate HBA in a H_2_O_2_ concentration-dependent manner, confirmed by the appearance of new aromatic proton peaks at 6.8 and 7.2 ppm. In the presence of equimolar concentration of H_2_O_2_ (1 mM), a majority of boronic esters were cleaved within 30 min, with a half-life of hydrolysis of ~5 min. Nearly all of boronic ester groups were cleaved by 5-fold excess of H_2_O_2_ (5 mM) within 5 min. However, in the absence of H_2_O_2_, the boronic ester remained intact even after 3 days. It was also determined that BRAP undergoes H_2_O_2_-triggered hydrolysis with a second-order rate constant of 1.67 (Lmol^−1^ s^−1^), which is constant with those of substituted phenylboronates^26^.

**Figure 2:**
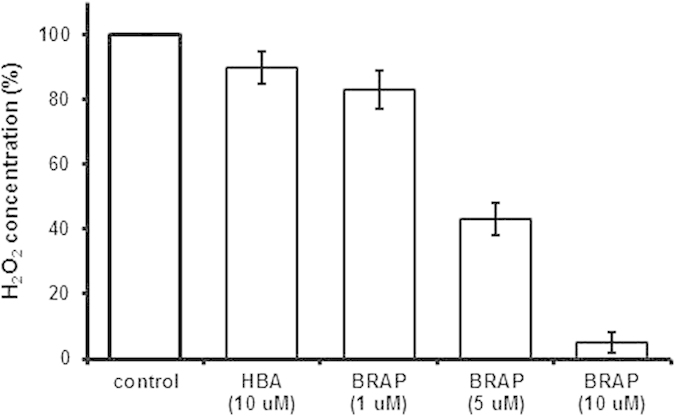
H_2_O_2_-scavenging ability of BRAP. H_2_O_2_ solution (10 μM) was mixed with HBA or BRAP for 10 min and the level of H_2_O_2_ was measured by Ample red assay. (n = 4).

It was hypothesized that BRAP could scavenge H_2_O_2_ during its H_2_O_2_-mediated boronate oxidation. We therefore investigated the ability of BRAP to scavenge H_2_O_2_ using Amplex Red assay. The addition of BRAP resulted in significant reduction in the concentration of H_2_O_2_, in a concentration-dependent manner (Fig. 2). A majority of H_2_O_2_ was scavenged by the same concentration of BRAP within 10 min. In contrast, HBA alone (10 μM) marginally reduced the concentration of H_2_O_2_. These observations demonstrate that BRAP readily reacts with H_2_O_2_ to render efficient elimination of H_2_O_2_.

### Antioxidant and anti-inflammatory activities of BRAP *in vitro*

We first performed LC-MS/MS analysis to confirm if BRAP reacts with H_2_O_2_ to generate HBA in cells. Murine macrophage cell line RAW264.7 cells were activated with H_2_O_2_ for 24 h to induce the further generation of ROS and then cell culture medium was replaced with fresh medium. BRAP (1 mM) was added to non-activated or activated cells and the cell lysates were analyzed with LC-MS/MS. As shown in Supplementary Fig. S2, the generation of HBA in activated cells was directly confirmed by the appearance of a peak in the LC trace identical to the standard HBA. However, no peak was observed with non-activated cells, demonstrating the BRAP is readily taken up by cells and generates HBA in the presence of intracellular ROS such as H_2_O_2_.

**Figure 3:**
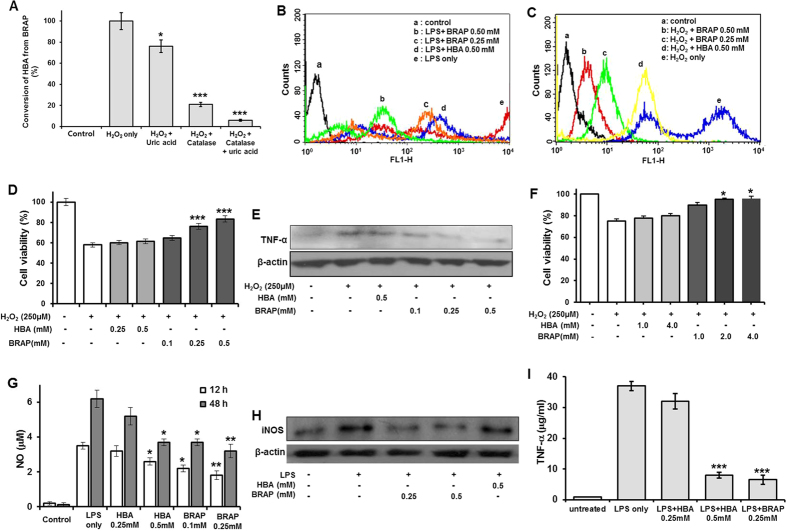
Antioxidant and anti-inflammatory effects of BRAP *in vitro*. (**A**) Conversion of BRAP into HBA in stimulated cells. Values are mean ± s.d. (n = 3). Inhibitory effects of BRAP on the generation of intracellular ROS in RAW264.7 cells stimulated by 1 μg/mL of LPS (**B**) and 250 μM of H_2_O_2_ (**C**). The generation of ROS was monitored by flow cytometry as an indicator of DCF fluorescence. Fluorescence was analyzed in 10,000 cells with excitation at 480 nm and emission at 530 nm. Data are presentative of three independent experiments. (**D**) Effects of BRAP on the viability of RAW264.7 cells activated with H_2_O_2_. ***p < 0.001 vs H_2_O_2_. (n = 4). (**E**) Inhibitory effects of BRAP on the expression of TNF-α in activated RAW264.7 cells. Data are presentative of three independent experiments. (**F**) Protective effects of BRAP on H_2_O_2_-stimulated adult rat cardiomyocytes. *p < 0.05 vs H_2_O_2_. (n = 4). (**G**) Inhibitory effects of BRAP on nitric oxide (NO) generation in LPS-stimulated RAW 264.7 cells. *p < 0.05, **p < 0.01 vs LPS. (n = 4). (**H**) Inhibitory effects of BRAP on the expression of *i*NOS in LPS-stimulated RAW 264.7 cells. Data are presentative of three independent experiments. (**I**) Inhibitory effects of BRAP on the generation of TNF-α in LPS-stimulated RAW 264.7 cells. ***p < 0.001 vs LPS. (n = 4).

Another set of LC-MS/MS experiments was performed to further investigate the H_2_O_2_-mediated conversion of BRAP to HBA in stimulated cells (Fig. 3A). We measured the level of cellular conversion of BRAP into HBA in the presence of catalase and uric acid. Catalase and uric acid were used as a scavenger of H_2_O_2_ and peroxynitrite, respectively^27^. The addition of catalase (200 μg) or uric acid (250 μM) attenuated significantly the conversion of BRAP into HBA. Conversion of BRAP into HBA was almost completely attenuated in cells in the presence of both catalase and uric acid. However, H_2_O_2_-scavenging catalase attenuated the cellular conversion of BRAP to HBA to a greater extent than peroxynitrite scavenging uric acid, suggesting BRAP preferentially reacts with H_2_O_2_ to generate HBA.

We next examined the antioxidant activities of BRAP using RAW264.7 cells. After stimulation by LPS (lipopolysaccharide), intracellular ROS production was analyzed by flow cytometry using DCFH-DA (dihydrodichlorofluorescein-diacetate) which can be oxidized by the action of various intracellular oxidants such as H_2_O_2_, peroxynitrite, and hydroxyl radical to become fluorescent DCF (dihydrodichlorofluorescein) (Fig. 3B; Supplementary Fig. S3A)^13^,^28^,^29^. Untreated cells have no significant DCF fluorescence. On the other hand, strong DCF fluorescence was observed in the cells treated with exogenous LPS because LPS induced the generation of ROS which oxidized DCFH-DA to fluorescent DCF. HBA at 0.5 mM showed moderate inhibitory effects on ROS generation, as demonstrated in previous studies^12^,^13^. However, 0.25 mM of BRAP exerted significantly stronger inhibitory effects on the LPS-induced ROS generation than 0.5 mM of HBA.

Stimulation with H_2_O_2_ for 8 h also induced a large generation of intracellular ROS, evidenced by remarkable rightward shift of DCF fluorescence (Fig. 3C; Supplementary Fig. S3B). However, BRAP treatment significantly inhibited the cellular generation of ROS in a concentration dependent manner. BRAP at 0.5 mM showed more suppressive effects on intracellular ROS generation than 0.5 mM of HBA, suggesting the higher antioxidant effects of BRAP than HBA. Stimulation with H_2_O_2_ also induced almost 50% of cell death at 24 h. HBA showed no inhibitory effects on H_2_O_2_-mediaed cell death. However, BRAP significantly suppressed cell death in H_2_O_2_-stimulated cells, in a dose dependent manner (Fig. 3D). H_2_O_2_-stimulation also enhanced the expression of pro-inflammatory cytokine, TNF-α (tumor necrosis factor-alpha). HBA exerted anti-inflammatory activity by suppressing the expression of TNF-α to some extent. However, BRAP exerted higher anti-inflammatory activity than HBA (Fig. 3E; Supplementary Fig. S4).

Since cardiovascular disease is one of the most common and clinically relevant problems associated with oxidative stress injury, we further examined the effect of BRAP on cellular protection from the H_2_O_2_-induced cell death using adult rat ventricular cardiomyocytes (ARVC) *in vitro*. We found that H_2_O_2_ (0.25 mM) resulted in approximately 30% cell death. Compared to the vehicle control, HBA showed no protective effects, even at 4 mM. However BRAP showed significant protection from H_2_O_2_-induced cell death in a concentration dependent manner (Fig. 3F).

Nitric oxide is one of major sources of oxidative stress and a well-known pro-inflammatory mediator in the pathogenesis of inflammation^14^,^30^. The effects of BRAP on the generation of nitric oxide in LPS-treated RAW264.7 cells were studied using a colorimetric assay based on the Griess reaction^13^,^31^. LPS induced a large amount of nitric oxide production and BRAP exerted the inhibitory effects on nitric oxide production in time- and dose-dependent manners (Fig. 3G). HBA alone also inhibited nitric oxide production, but only at a concentration higher than 0.5 mM, suggesting superior antioxidant activity of BRAP. In order to confirm whether the inhibitory effects of BRAP on nitric oxide generation results from the suppression of *i*NOS (inducible nitric oxide synthases), LPS-stimulated cells were subjected to immunoblotting with an antibody of *i*NOS (Fig. 3H; Supplementary Fig. S5). LPS-induced expression of *i*NOS was significantly suppressed by 0.5 mM of HBA. BRAP also exerted remarkable inhibitory effects on *i*NOS expression. Even 0.25 mM of BRAP almost completely prevented the up-regulation of *i*NOS and displayed more inhibitory effects than 0.5 mM of HBA. The level of nitric oxide is proportional to the expression of *i*NOS. However, we found that BRAP could inhibit the nitric oxide generation only 50%. This discrepancy can be explained by the different condition of cell culture. For the detection of nitric oxide using Griess reagents, cells were cultured in phenol red-free medium without FBS.

We also investigated the anti-inflammatory effects of BRAP on LPS-stimulated cells by measuring the level of inflammatory cytokines such as TNF-α (Fig. 3I). The level of TNF-α was significantly increased by the LPS treatment. BRAP significantly suppressed the level of TNF-α. HBA alone also reduced the expression of TNF-α, but only at a higher dose than BRAP. These observations from the proof-of concept experiments support that rationally designed BRAP is able to scavenge overproduced H_2_O_2_ and serves as a H_2_O_2_-activatable antioxidant. It can be also reasoned that the enhanced antioxidant activity of BRAP is contributed to the combined effects of H_2_O_2_-scavenging boronic esters and therapeutic HBA released after H_2_O_2_-mediated boronate oxidation.

Initial cytotoxicity experiments of BRAP demonstrated no significant cytotoxicity at concentrations as high as 4 mM after 24 h of incubation in various cell lines and primary culture of ARVC *in vitro* (Supplementary Fig. S6).

### Therapeutic efficacy of BRAP in hepatic I/R injury

**Figure 4:**
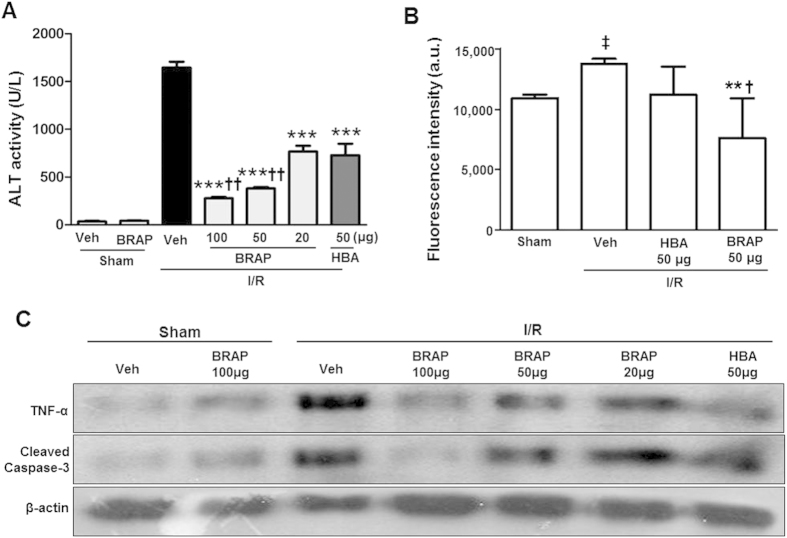
Therapeutic effects of BRAP in hepatic I/R injury. (**A**) Reduction of the ALT activity by BRAP. ***p < 0.001 vs Veh, ^††^*p* < 0.01 vs HBA. (n = 4). (**B**) The level of H_2_O_2_ in liver tissues determined by Amplex Red assay. **p < 0.01 vs Veh, ^†^*p* < 0.05 vs HBA. ^‡^*p* < 0.05 vs sham. (n = 3). (**C**) Western blot assay of TNF-α and cleaved caspase-3 in the liver tissues. Actin protein expression was used as an internal control for protein loading. Data are representative of three independent experiments. Hepatic I/R injury was induced by 1 h ischemia followed by 12 h reperfusion.

We investigated whether BRAP could reduce ROS generation and inhibit apoptosis in a mouse model of hepatic I/R injury. I/R was induced first by 1 h of ligation of hepatic artery and portal vein. Then, only hepatic artery was reperfused, which would achieve I/R injury to approximately 70% of the liver in the right lower lobe. This method of partial ischemia prevents mesenteric venous congestion by allowing portal decompression throughout the right and caudate lobes of the liver and has been widely used in liver I/R model^32^,^33^,^34^. BRAP (25, 50 or 100 μg) or HBA (50 μg) was then administrated intraperitoneally (*i.p*) at the time of reperfusion. I/R induced liver damages as evidenced by the increase in alanine transaminase (ALT) in the serum (Fig. 4A). HBA at a dose of 50 μg significantly reduced the ALT activity. BRAP also significantly suppressed the ALT activity in a concentration-dependent manner, but showed stronger protective effects than HBA. We investigated the inhibitory effects of BRAP on the generation of H_2_O_2_ in the liver of mice undergoing hepatic I/R injury. As shown in Fig. 4B, the level of H_2_O_2_ in the liver was elevated by I/R injury and 50 μg of HBA showed inhibitory effects, but non-significantly. However, the same dose of BRAP significantly reduced the level of H_2_O_2_, demonstrating the ability of BRAP to scavenge H_2_O_2_ *in vivo*.

Anti-apoptotic activities of BRAP was evaluated by measuring the level of caspase-3, a marker of apoptosis. HBA had minimal effects on the cleaved caspase-3. On the other hand, the same dose of BRAP significantly inhibited the cleavage of pro-caspase 3. BRAP also remarkably suppressed the expression of pro-inflammatory TNF-α (Fig. 4C; Supplementary Fig. S7). These results demonstrate that BRAP exerts anti-inflammatory and anti-apoptotic activity during hepatic I/R injury of mice. LC-MS/MS analysis of the liver tissues was also performed to further confirm that BRAP could target the site of I/R injury and react with H_2_O_2_ to generate HBA in liver. As shown in Supplementary Fig. S8, the liver of healthy mice treated with BRAP has no HBA peak in the LC trace. However, HBA was detected in the liver of mice which was undergoing I/R injury and was also treated with BRAP. No HBA detection was observed in lung, heart, kidney and spleen of mice undergoing hepatic I/R injury. These observations demonstrate that BRAP targets the hepatic I/R injury and generates HBA from the H_2_O_2_-mediated boronate oxidation in liver.

**Figure 5:**
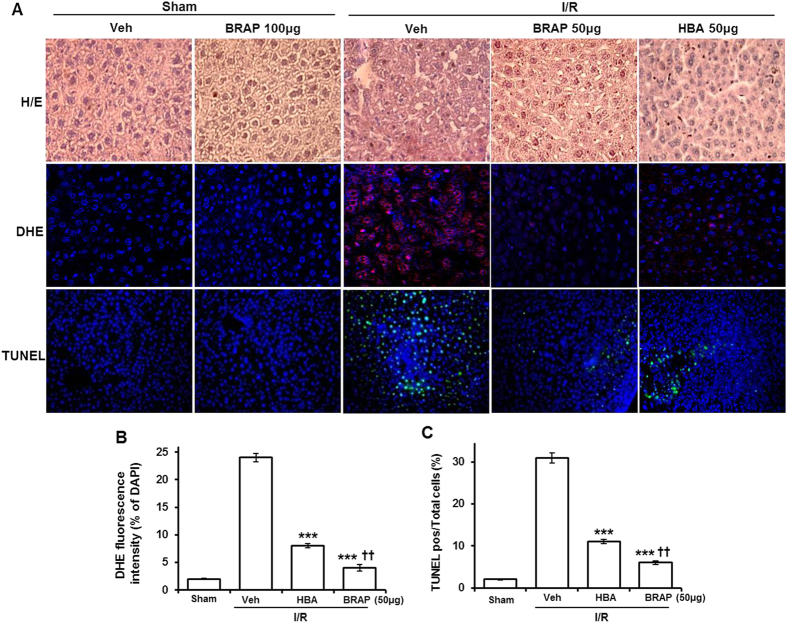
Histological examination of liver tissues of mice undergoing hepatic I/R. (**A**) Representative confocal microscopy images of H&E, DHE and TUNEL staining of liver tissues. (**B**) Quantification of the DHE fluorescent signal in the liver tissues. **(C)** Quantification of TUNEL positive cells/total cells in the liver tissues. ***p < 0.001 vs Veh, ^††^*p* < 0.01 vs HBA. (n = 3).

Histological analysis demonstrated that severe hepatic damages were induced by I/R, as evidenced by disruption of tissue architecture, hepatic necrosis randomly distributed through the parenchyma and increased leukocyte infiltration (Fig. 5A). Features of apoptosis were also observed, appearing as cell shrinkage, chromatin condensation, dense chromatin fragmentation and formation of apoptotic bodies^35^,^36^. HBA showed minimal therapeutic effects on hepatic damages during I/R, but the same dose of BRAP resulted in significant attenuation of tissue damages. In order to investigate the effects of BRAP on oxidative stress during I/R injury, tissues were stained with dihydroethidium (DHE), which is permeable into cells and becomes fluorescent in the presence of oxidants such as superoxide. I/R injury significantly increased the oxidative stress, evidenced by the strong purple DHE fluorescence. The level of oxidative stress was suppressed by both HBA and BRAP, but BRAP exhibited higher inhibitory effects on ROS generation than HBA (Fig. 5B). The addition of selective superoxide scavenger, polyethylene glycolated superoxide dismutase (PEG-SOD) remarkably suppressed the DHE fluorescent signal staining, suggesting that DHE staining is specific for superoxide (Supplementary Fig. S9).

TUNEL (Terminal deoxynucleotidyl transferased UTP nick end labelling) staining was performed to investigate the effects of BRAP on apoptotic cell death because it is recently recognized that mediators of apoptosis are activated during I/R and post-ischemic cell death is caused by apoptosis^37^,^38^. I/R injury caused severe hepatic apoptosis, confirmed by the number of strong TUNEL positive cells, which is consistent with other studies reporting that around 15% of apoptotic hepatocytes was measured by TUNEL staining after 45 min of ischemia and 24 h of reperfusion^32^. HBA exhibited modest inhibitory effects on hepatic apoptosis, whereas the same dose of BRAP showed significantly greater anti-apoptotic effects than HBA (Fig. 5C).

### Therapeutic efficacy of BRAP in cardiac I/R injury

**Figure 6:**
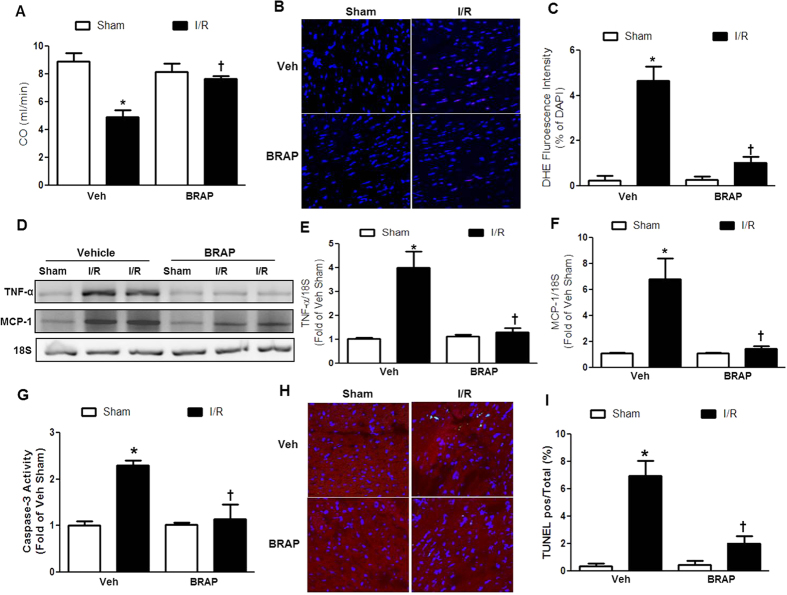
Therapeutic effects of BRAP on cardiac function after I/R. (**A**) Cardiac Output (CO) after BRAP administration for 2 weeks after I/R injury. *p < 0.05 vs sham of each group, ^†^*p* < 0.05 vs Veh I/R. (n = 4). (**B**) Representative confocal microscopy images of DHE stained cardiomyocytes. (**C**) Quantification of DHE fluorescent signal in the tissue. *p < 0.05 vs sham of each group, ^†^*p* < 0.05 vs Veh I/R. (n = 3). (**D**) Representative images of mRNA expression of inflammatory markers (TNF-α and MCP-1). 18S mRNA expression was used as an internal control. Data are presentative of three independent experiments. Quantification of the mRNA expression of caspase-3 activity (**E**) and MCP-1 (**F**). *p < 0.05 vs sham of each group, ^†^*p* < 0.05 vs Veh I/R. (n = 3). (**G**) Quantification of caspase-3 activity after BRAP administration. *p < 0.05 vs sham of each group, ^†^*p* < 0.05 vs Veh I/R (n = 4). (**H**) Representative confocal microscopy images of TUNEL staining of cardiomyocytes. (**I**) Quantification of TUNEL positive cardiomyocytes/total cells. *p < 0.05 vs sham of each group, ^†^*p* < 0.05 vs Veh I/R (n = 3). DHE staining, mRNA expression measurement, caspase activity assay and TUNEL analysis were done in heart tissue 24 h after cardiac I/R injury.

The beneficial effects of BRAP were further investigated using a mouse model of cardiac I/R injury. After 45 min of ischemia, BRAP (1.5 mg/kg) or vehicle was administered *i.p*. at the time of reperfusion, and then daily (1.5 mg/kg/day) for 2 weeks. For cardiac functional analysis, we performed pressure-volume (PV) loop measurement and echocardiography at 2 weeks after I/R surgery. PV loop analysis showed a significant reduction of cardiac output (CO), stroke work (SW) and ejection fraction (EF) 2 weeks after I/R injury (Fig. 6A; Supplementary Fig. S10A,B). Additional cardiac functional analysis using echocardiography also showed significant decrease in fractional shortening (FS) at 2 weeks after I/R surgery (Supplementary Fig. S10C). Administration of BRAP significantly attenuated I/R-induced cardiac dysfunction both in PV loop analysis and echocardiography.

We next determined whether the benefits of BRAP after I/R were associated with attenuation of oxidative stress, inflammation, and apoptosis. These mechanistic analyses were performed in heart tissues 24 h after cardiac I/R injury. The generation of superoxide was significantly increased after I/R in vehicle-treated mice (Fig. 6B,C). BRAP administration significantly decreased DHE staining, demonstrating the beneficial antioxidant effects of BRAP on I/R. In addition, evaluation of inflammatory responses showed that I/R injury significantly increased mRNA levels of TNF-α and monocyte chemotactic protein-1 (MCP-1). BRAP administration significantly decreased the expression of TNF-α and MCP-1 compared with vehicle-treated mice (Fig. 6D–F). To assess the anti-apoptotic effects of BRAP after I/R, caspase-3 activity assay and TUNEL staining were performed. Caspase-3 activity was significantly increased after I/R (Fig. 6G). BRAP effectively inhibited caspase-3 activation. In addition, I/R induced the greater extent of cardiomyocyte apoptosis as demonstrated by significant increase in TUNEL-positive cardiomyocytes after 24 h of I/R (Fig. 6H,I) which was effectively inhibited by BRAP. These results demonstrate that BRAP treatment effectively prevented I/R-induced cardiac damages by blocking oxidative stress and inflammation, resulting in inhibition of apoptosis.

### Toxicity studies

**Figure 7:**
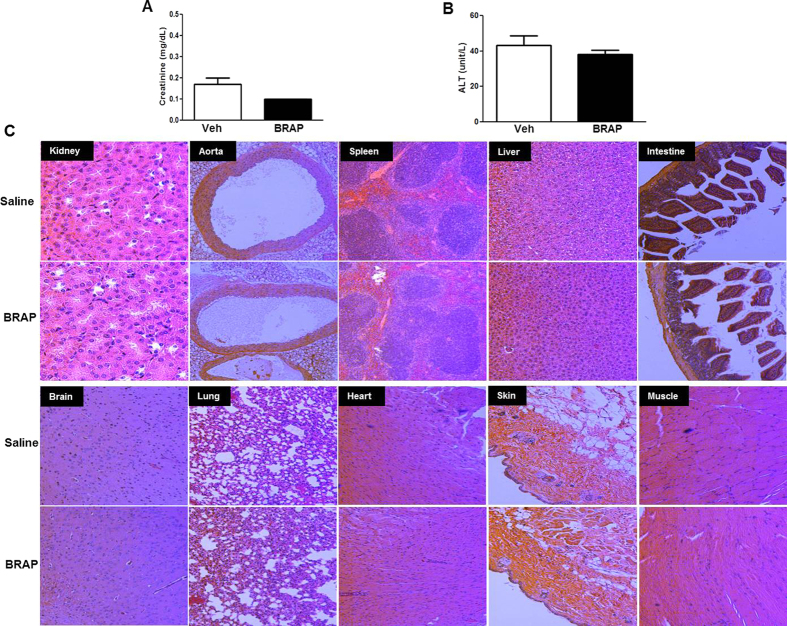
Safety profile of BRAP (1.5 mg/kg/day) after daily intraperitoneal administration for 7 days. (**A**) The level of creatinine. BRAP has a too low statistical error bar to see. (**B**) The level of ALT. (**C**) Representative H/E stained tissue sections of different organs. Organs were collected 7 days after daily BRAP administration. (n = 4).

To determine the potential cumulative toxic effects of BRAP, we administered BRAP (1.5 mg/kg/day) daily for 7 days in mice. Serum tests for renal and hepatic functions showed no significant abnormalities after 7 days (Fig. 7A,B). In addition, there was no significant histological evidence of accumulated toxicity in the different organs after receiving BRAP for 7 days (Fig. 7C). The results of this initial toxicity study reveal that BRAP has excellent safety profiles even at the therapeutic dose *in vivo*.

## Discussion

The strategy employed for the development of H_2_O_2_-activatable antioxidant prodrug uses phenylboronic ester groups that can be readily cleaved by H_2_O_2_ to generate phenol groups. Boronic ester has been widely used as a self-immolative protecting group in the development of molecular sensors, drugs and drug delivery systems due to its excellent specific reactivity to H_2_O_2_ and nontoxicity^3^,^4^,^23^,^39^,^40^,^41^. As shown in Fig. 1A, we synthesized H_2_O_2_-activatable BRAP, (4-(5-(hydroxymethyl)-5-methyl-1,3,2-dioxaborinan-2-yl)phenyl) methanol. This strategy would allow BRAP to exert a dual mode of therapeutic actions. First, BRAP is specifically oxidized by high level of H_2_O_2_, subsequently limiting H_2_O_2_-mediated oxidative stress and injuries. Second, H_2_O_2_-mediated boronate oxidation results in the generation of free HBA, which exerts its intrinsic antioxidant and anti-inflammatory activities in the tissues undergoing oxidative stress.

Peroxynitrite is a strong biological oxidant with an extremely short half-life of <10 ms and could have deleterious effects on a wide variety of biomolecules such as nucleic acids, proteins and lipids, leading to cell apoptosis or necrosis^42^. Several studies have reported that peroxynitrite stoichiometrically reacts with aromatic boronates a million times faster than H_2_O_2_ 26. However, it has been recently reported that peroxynitrite reacts predominantly with carbon dioxide in cells to produce highly strong and short-lived radicals, carbonate radical and nitrogen peroxide that have significantly different chemistries^43^. Ferrer-Sueta *et al.* reported that carbon dioxide at a physiological concentration (≥1.3 mM) is twice as effective as 20 μM of boronate at trapping peroxynitrite and a majority of biological reaction of oxidant sensitive probes for peroxynitrite are mediated by carbonate radicals and nitrogen peroxide^44^. Moreover, peroxide and peroxynitrite could not be easily distinguished. In this regard, we studied mainly the reactivity of BRAP to H_2_O_2_ which is highly stable and one of the most abundant ROS in I/R injury.

H_2_O_2_ produced during I/R plays an important role by releasing pro-inflammatory cytokines and inducing apoptosis, which further exacerbates tissue damages^45^. Thus, minimizing tissue damages is the most important aspect of preserving organ functions and decreasing morbidity and mortality^9^,^46^. However, the beneficial effects of general antioxidant therapy in human clinical studies have been disappointing^47^,^48^. There could be a number of explanations for this finding, such as lack of complete ROS inhibition, non-specific suppression of ROS or poor trial design. Although overproduction of H_2_O_2_ (in μM) during I/R injury is deleterious, H_2_O_2_ at very low levels (in nM) has been shown to be essential for cellular signaling for normal physiological cellular functions^49^. Our approach based on H_2_O_2_-activatable BRAP will allow effective lowering of H_2_O_2_ level only when there is overproduction of H_2_O_2_ and spare general H_2_O_2_ suppression in a normal physiological setting. Thus, our targeted strategy will not only be more effective but will also limit undesirable potential side effects.

An ideal targeted drug will exhibit desired pharmacological effects with temporal and spatial control of therapeutic activities. The goals of the targeted therapy are to have target area specificity and stimulus-sensitivity, which will enhance the effectiveness of the drug as well as simultaneously decrease the undesirable side effects^50^. Although most ROS are extremely short lived, H_2_O_2_ is highly stable. Consequently, the concentration of H_2_O_2_ tends to accumulate in high level during oxidative stress resulting in cellular damages. For these reasons, H_2_O_2_ is an attractive target for the targeted drug therapy for oxidative stress. The ability of BRAP to react with H_2_O_2_ will allow it to be activated specifically by a pathologic overproduction of H_2_O_2_, as seen during I/R injury.

Empirical use of natural products and synthesis of their derivatives have been used for the development of new therapeutic drugs^51^. HBA, one of major active components of *Gastrodia elata,* plays protective roles against brain ischemic injury and coronary artery diseases^52^. We developed BRAP as a H_2_O_2_-activatable prodrug of HBA by exploiting H_2_O_2_-mediated boronate oxidation as a chemoselective approach to react with and scavenge H_2_O_2_ in complex biological systems. BRAP with a self-immolative boronic ester protecting group is able to scavenge H_2_O_2_ and release therapeutic HBA. This property allows BRAP to exert highly potent therapeutic effects in tissues that are undergoing oxidative stress in a targeted manner. In this study, BRAP displayed remarkable protective actions on hepatic and cardiac I/R injury at a dose of less than 5 mg/kg. However, the same dose of HBA showed marginal therapeutic actions. In previous studies using mouse models of ischemic brain injury, HBA exerted sufficient therapeutic effects at doses of more than 25 mg/kg^17^,^19^,^21^. Given its lower effective dose and superior therapeutic activity of BRAP, BRAP holds great potential as a therapeutic agent for the treatment of H_2_O_2_-associated diseases.

In addition, masking of HBA by a boronic ester bond also makes it biocompatible and water soluble, which provides great benefits in pharmaceutical applications. However, further optimization, including administration routes, doses, pharmacokinetics and long-term toxicity are necessary to maximize the full potential of BRAP for clinical settings.

To conclude, we have developed H_2_O_2_-activatable antioxidant BRAP as an I/R targeted therapeutic agent. BRAP with a self-immolative boronic ester protecting group exerted highly potent antioxidant, anti-inflammatory and anti-apoptotic activities in the site of I/R injuries due to the synergistic effects of H_2_O_2_-scavenging boronic esters and therapeutic HBA. These findings, we believe, will provide foundation for developing a potential drug therapy that will minimize side effects while being able to preferentially target the area that is affected during I/R. We anticipate that BRAP could be a novel and effective therapeutic option in various oxidative stress-associated conditions, and will have great potential in pharmaceutical applications.

## Methods

### Synthesis of BRAP

(4-(Hydroxymethyl)phenyl)boronic acid (1.50 g) and 2-(hydroxymethyl)-2-methylpropane-1,3-diol (1.18 g) were added into 20 mL of dry tetrahydrofuran under nitrogen and the mixture was allowed for at room temperature with mechanical stirring. When the reaction mixture became clear after 24 h of reaction, 0.2 g of Na_2_SO_4_ was added. The reaction was allowed for at room temperature overnight and the solvent was evaporated using a rotary evaporator. BRAP was obtained using silica gel chromatography (hexane/ethyl acetate = 70/30). BRAP was characterized using NMR (JNM-ECA600 JEOL, Peabody, MA) and GC-MS (HP6890 Series GC System, Agilent, Agilent Technologies, Willington). ^1^H NMR (400 MHz, CDCl_3_): *δ* 7.7(*m*, 2 H), 7.3(*m*, 2 H), 4.6 (m, 2 H, Ar-CH_2_OH), 3,4 (m, 2 H, CCH_2_OH), 0.8(m, 3 H, CCH_3_); ^13^C NMR (400 MHz, CDCl_3_): *δ* 144.5, 144.0, 127.5, 67.0, 64.5, 63.5, 42.0, 16.3; GC-MS (m/z):[M^+^] calc. for C_12_O_4_H_17_B, 236.066; found 235.141. Elemental Analysis (calc, found for C_12_O_4_H_17_B): C (61.08, 60.60), H (7.25, 7.29).

### Cell culture

RAW 264.7 cells and mouse hepatocytes were obtained from Korean Cell Line Bank (Seoul, Korea) and cultured in DMEM containing 10% FBS (fetal bovine serum) with 1% penicillin/streptomycin. Cells with passage numbers less than 20 were used. All cells were cultured in an incubator with 5% of CO_2_ at 37 °C.

### Cytotoxicity and H_2_O_2_ scavenging of BRAP

3-(4,5-Dimethylthiazil-2yl)-2,5-diphenyltetrazolium bromide (MTT) assay was performed to evaluate the cytotoxicity of BRAP. Cells were cultured in a 24 well plate (Nunc™ Cell Culture plate) for 24 h to reach ~80% confluency. Cells were treated with various amount of BRAP and incubated for 24 h. Each well was given 100 μL of MTT solution and was incubated for 4 h. Two hundred microlitters of dimethyl sulfoxide (DMSO) was added to each well to dissolve the resulting formazan crystals. After 10 min incubation, the absorbance 570 nm was measured using a microplate reader (Biotek Instruments, Winooski, VT). The cell viability was determined by comparing the absorbance of BRAP treated cells that of control cells.

The ability of BRAP to scavenge H_2_O_2_ was evaluated be measuring the concentration of H_2_O_2_ after incubation with BRAP. After the addition of BRAP (1, 5 or 10 μM) or HBA (10 μM) to 1 mL of H_2_O_2_ solution (10 μM) for 10 min, the concentration of H_2_O_2_ was determined using the Amplex Red assay (Invitrogen, Carlsbad, CA) according to the manufacturer’s protocol.

### LC-MS/MS analysis of cell lysates

RAW264.7 cells were seeded in a T-75 flask containing 10 mL of medium and allowed to attach for 24 h. Cells were stimulated with 250 μM of H_2_O_2_ for 12 h. Then, cells were washed with new culture medium and treated with 1 mM of BRAP for 12 h. Cells were washed with new culture medium and cell pellets were added with 100 μL of methanol. The mixture was vortexed and then additional 900 μL of methanol was added. The contents were thoroughly mixed by vortexing and high molecular materials were removed by sequential centrifugation (10,000 × *g*) for 10 min. The supernatant was immediately stored at −80 °C until analysis. Upon analysis, 5 μL was injected and the peak for HBA was analyzed using a LC-MS/MS spectrometer (6410 Triple Quad LC /MS/MS, Agilent Technologies, Willington) equipped with a column (Synergi 4 μ Hydro RP 80A, 150 × 2.00 mm). The mobile phase components were: A = water, 0.1% formic acid; B = acetonitrile, 0.1% formic acid. HBA was eluted according to the linear gradient from 5% B buffer to 100% at a flow rate of 0.25 mL/min. Multiple reaction monitoring (MRM) was conducted by *m/z* 124→105 for HBA. Ions were generated in negative ionization mode using electrospray ionization interface. The fragmenter potential was set to 50 V and the collision energy was set to 10.

### Flow cytometry

RAW 264.7 cells (4 × 10^5^) were cultured in a 24 well culture plate for 24 h and treated with LPS (1 μg/mL) or H_2_O_2_ (250 μM). Cells were treated with HBA or BRAP for 1 h and then stimulated with LPS for 1 h or H_2_O_2_ for 8 h. For the detection of ROS, DCFH-DA (Sigma-Aldrich, St. Louis, MO) was used as ROS probe. The cell suspensions (1 mL) were transferred to a 5 mL culture tube containing PBS and added with 5 μM of DCFH-DA dissolved in DMSO, followed by gentle mixing. The cells were incubated for 15 min at 37 °C in the dark and added with 400 μL of 1× binding buffer. The stained cells were analyzed by flow cytometry (FACS Caliber, Becton Dickinson, San Jose, CA). A total of 1.0 × 10^4^ events were counted for each sample.

### Detection of nitric oxide

RAW 264.7 cells (4 × 10^5^ cells/well in a 24 well plate) were cultured in phenol red-free medium without FBS and pretreated with 3 μL of LPS (1 mg/mL) in the presence of various amounts of BRAP for 4 h. Cell culture medium was replaced with fresh medium and cells were incubated for 8 h. The concentration of nitric oxide was determined using a colorimetric assay based on the Griess reaction. Fifty microlitters of cell culture medium was collected and given 100 μL of Griess reagent (6 mg/mL) at room temperature for 10 min, and then the nitric oxide concentration was determined by measuring the absorbance at 570 nm using a microplate reader (Synergy MX, BioTek Instruments, Inc, Winooski, VT). The standard curve of nitric oxide was constructed using known concentrations of sodium nitrite. Untreated cells were used as negative control.

### Immunoblot analysis

Cells (2 × 10^6^/well) cells were treated with various concentrations of BRAP 24 h in the presence of LPS (1 μg/mL) or H_2_O_2_ (250 μM) and then washed with fresh PBS twice. Proteins were extracted from the cells using a RIPA buffer (Thermo Scientific, Rockford, IL) on ice and the protein content was measured using BCA assay. Electrophoresis was performed using 20 μg of cell lysate on a 10% polyacrylamide gel under the same conditions and proteins were transferred to PVDF membranes (Millipore, Billerica, MA). The blot was incubated with *i*NOS monoclonal antibody or TNF-α monoclonal antibody (Santa Cruz Biotechnology, Dallas, TX) at a 1:1000 dilution ratio and HRP-conjugated anti-goat (Millipore, Billerica, MA) which is used as a secondary antibody. Actin protein expression was used as an internal control for protein loading. Immunoblot signals were developed using SuperSignal Ultra chemiluminescent reagent (Pierce, Rockford, IL). Images have been cropped for presentation. Full-length images of blots are presented in Supplementary Information.

### Animal surgeries

Hepatic I/R surgery was performed in 12 week-old male mice as described previously^10^. Briefly, mice (8 weeks old and 20 g, Balb/c, Orient Bio, Korea) were anaesthetized with intraperitoneal injection of mixed solution of ketamine and Xylazine (8:1 ratio), and midline incision was performed for laparotomy. After identifying the portal triad and biliary tree, the main trunk of hepatic artery and portal vein were clamped with vascular clip except for the vasculatures to the right lower lobe to achieve ischemic injury to approximately 70% of the liver. After 1 h of ischemia, reperfusion was achieved by releasing the vascular clip. No vascular clamp was done for the sham group of mice. After 1 h, reperfusion was achieved by releasing the vascular clip. Immediately, BRAP (25, 50 or 100 μg) or HBA (50 μg) was intraperitoneally given to mice. The same amount of saline was given to sham groups. Then, the incision was closed with 5–0 black silk suture. For LC-MS/MS analysis, 4 h after reperfusion, liver, lung, heart, kidney and spleen were removed and homogenized on ice. The tissue lysates were analyzed for detection of HBA using a LC-MS/MS spectrometer (6410 Triple Quad LC /MS/MS, Agilent Technologies, Willington) equipped with a column (Synergi 4 μ Hydro RP 80A, 150 × 2.00 mm). All the animal experiments were carried out according to the guidelines of the institution animal ethical committee of Chonbuk National University, Korea.

Cardiac I/R surgery was performed in 12 week-old male mice as described previously^53^. Briefly, mice were anaesthetized with intraperitoneal injection of mixed solution of ketamine and Xylazine (8:1 ratio), and placed on a rodent ventilator (model 687, Harvard Respirator). After thoracotomy, the left anterior descending artery (LAD) artery was identified and ligated with a 7–0 silk suture tied around a specialized 30G-catheter. The animal remained under anesthesia and ventilation for 45 min of ischemia, after which reperfusion was achieved by cutting the suture and re-establishing arterial perfusion. Sham operated mice underwent the same procedure without LAD occlusion. All experimental procedures were approved by the Institutional Animal Care and Use Committee of Beth Israel Deaconess Medical Center.

### Amplex red assay of liver tissue

Liver tissues were randomly collected in PBS with the same weight/volume ration and homogenized using a homogenizer (PRO Scientific, PRO 200). Tissue homogenates were centrifuged at 1,000 × *g* at 4 °C for 10 min and the supernatants were collected. The supernatant was treated with Amplex Red assay reagents (Invitrogen, Carlsbad, CA) and incubated at room temperature for 30 min. The concentration of H_2_O_2_ in the supernatant was determined using a microplate reader (Synergy MX, BioTek Instruments, Inc, Winooski, VT) with excitation at 530 nm and emission at 590 nm.

### Cardiac functional analysis

Cardiac function was evaluated using the pressure-volume loop measurement and echocardiography 2 weeks after I/R as described previously^54^. Pressure-volume parameters were measured after isoflurane (2%) inhalant anesthesia using a 1.4 Fr. micro-tip pressure-volume catheter (ScisenseInc, Ontario, Canada) inserted into the right common carotid artery. The catheter was gently advanced into the left ventricle to obtain LV hemodynamic parameters. Data was recorded using a Powerlab system (ADInstruments, Colorado Springs, CO). Beat by beat pressure-volume parameters including heart rate (HR), stroke volume (SV), stroke work (SW) and cardiac output (CO) were measured and analyzed using CardioSoft Pro software (CardioSoft, Houston, TX). Transthoracic echocardiography was performed using a Vevo2100 ultra-high frequency small animal imaging system with MS400 transducer (18–38 MHz) (Visualsonics, Tronto, Canada).

### ALT activity assay

Livers were removed from the mice and homogenized in PBS. Total serum proteins were extracted from the tissue homogenate according to the manufacturer’s suggested protocol. The activity of serum ALT was determined with an ALT enzymatic assay kit (Asan Pharma, Seoul, Korea) using a microplate reader (Synergy MX, BioTek Instruments, Inc, Winooski, VT).

### Apoptosis assays

For liver apoptosis assay, terminal deoxynucleotidyl transferased UTP nick end labeling (TUNEL) staining was performed using a DeadEnd^TM^ Fluorometric TUNEL kit (Promega, Madsion, WI). Tissue sections were stained for nuclei (4′,6-diamidino-2-phenylindole (DAPI) staining) and apoptotic nuclei (TUNEL staining) and analyzed using a confocal laser scanning microscope. For heart tissues, TUNEL staining was performed using *in situ* cell death detection Kit (Roche Applied Science, Indianapolis, IN) as described previously. To distinguish cardiomyocyte from non-cardiomyocyte nuclei, we used triple stain for nuclei (DAPI staining), apoptotic nuclei (TUNEL staining), and cardiomyocytes (α-Actinin staining), and analyzed the stained sections using confocal microscopy. A minimum of ~10 high power fields with ~2000 nuclei/field were counted for each sample.

### RNA isolation and reverse transcription -polymerase chain reaction (RT-PCR)

The tissue samples were homogenized in TRIzol reagent (Life Technologies, Gaithersburg, MD) and total RNA was extracted from the tissue according to the manufacturer’s suggested protocol. Each sample was treated with DNase I (Gibco BRL, Rockville, MD) to eliminate any possible DNA contamination. Total RNA concentration was determined from spectrophotometric optical density measurement (260 and 280 nm). Reverse transcriptase reactions were then carried out using the RNA PCR Core Kit (PE Applied Biosystems, Foster City, CA). Each reaction tube contained 10 μg of total RNA in a volume of 150 μl containing 5 mmol/L MgCl_2_, 1 × PCR Buffer II, 500 μmol/L of each dNTP, 0.6 U/μl of RNase inhibitor, 2.5 U/μl of MuLV Reverse Transcriptase, 2.5 μmol/L of random hexamers and DEPC-treated water to volume. Reverse transcriptase reactions were carried out in a DNA Thermal Cycler 480 (Perkin Elmer, Branchburg, NJ) at 42 °C for 20 min and 99 °C for 5 min. The cDNA was then stored at −20 °C. mRNA expression levels were analyzed by RT-PCR with following specific primers: for pro-inflammatory factors, tumor necrosis factor-α (TNF-α) sense, 5′-CCT CAG CCT CTT CTC CTT CCT-3′, TNF-α antisense, 5′-GGT GTG GGT GAG GAG CA-3′, monocyte chemotactic protein-1 (MCP-1) sense, 5′-ACC TGC TGC TAC TCA TTC AC-3′, MCP-1 antisense, 5′-TTG AGG TGG TTG TGG AAA AG-3′, 18S sense, 5-GTT ATG GTT CCT TTG TCG CTC GCT C-3, 18S anti-sense, 5-TCG GCC CGA GGT TAT CTA GAG TCA C-3. PCR was performed using the Universal PCR Master Mix, 100 nmol/L of primers and various concentrations of RT product. Amplification conditions included 2 min at 50 °C and 10 min at 95 °C, and then run for 40 cycles at 95 °C for 15 sec and 60 °C for 1 min on the ABI PRISM 7700 sequence detection system (PE Applied Biosystems). Standard curves were constructed on a 1:2 serial dilution of DNA Template Reagent (PE Applied Biosystems). The threshold cycle, which represents the PCR cycle at which an increase in reporter fluorescence above background is first detected, was determined by the software, based on the generated standard curves. For RT-PCR ribosomal 18S primers acted as internal controls and all RT-PCR signals were normalized to the 18S signal of the corresponding RT products.

### Caspase-3 activity assay

The hearts were frozen in liquid nitrogen and homogenized in caspase lysis buffer (20 mM HEPES (pH 7.5), 10 mM KCl, 1.5 mM MgCl_2_, 1 mM EDTA, 1 mM EGTA, 1 mM DTT, 0.1 mM PMSF, 10 μg/ml leupeptin, 2 μg/ml aprotinin) for 15 min. on ice. They were then mechanically disrupted and centrifuged at 15,000 × *g* for 20 min to obtain cell lysates. Equal amounts of proteins were incubated with 50 μM of caspase substrate in caspase reaction buffer mM 50 HEPES (pH 7.4), 75 mM NaCl, 0.1% CHAPS, 2 mM DTT in a 96-well microplate at 37 °C for 1 h. The activity of caspase-3 was determined with colorimetric assay kit (R&D Systems, Minneapolis, MN) as described previously^53^,^55^. Briefly, protein samples were added to substrates of Acetyl-Asp-Glu-Val-Asp-*p*-nitroanilide. The enzyme-catalyzed release of *p*-nitroanilide was measured using a spectrometer at 405 nm.

### Reactive oxygen species (ROS) staining

The optimal cutting temperature (OCT)-embedded tissues were fixed in 4% paraformaldehyde. Tissue sections were incubated with 5 μM dihydroethidium at 37 °C for 30 min in a humidified chamber protected from light. Then, 4′,6-diamidino-2-phenylindole was applied. Images were acquired by confocal fluorescence microscope.

### Statistical analyses

Values were expressed as mean ± s.d. Comparisons between and within groups were conducted with unpaired Student t-tests and repeated-measures ANOVA using GraphPad Prism 5.0 (San Diego, CA), respectively. Probability (*p*) values of <0.05 were considered significant.

## Additional Information

**How to cite this article**: Lee, D. *et al.* Hydrogen peroxide-activatable antioxidant prodrug as a targeted therapeutic agent for ischemia-reperfusion injury. *Sci. Rep.* **5**, 16592; doi: 10.1038/srep16592 (2015).

## Supplementary Material

Supplementary Information
