# Supplementary material for: Hydrogen peroxide-activatable antioxidant prodrug as a targeted therapeutic agent for ischemia-reperfusion injury
Source: Sci Rep. 2015 Nov 13;5:16592. doi: 10.1038/srep16592 (PMC4643254; doi:10.1038/srep16592)
Supplement: Supplementary Information [file srep16592-s1.pdf]

Supplementary Information

Hydrogen peroxide-activatable antioxidant prodrug as a targeted therapeutic agent for ischemia-reperfusion injury

Dongwon Lee, Seunggyu Park, Soochan Bae, Dahee Jeong, Minhyung Park, Changsun Kang
Wooyoung Yoo, Mohammed A. Samad, Qingen Ke, Gilson Khang, Peter M. Kang

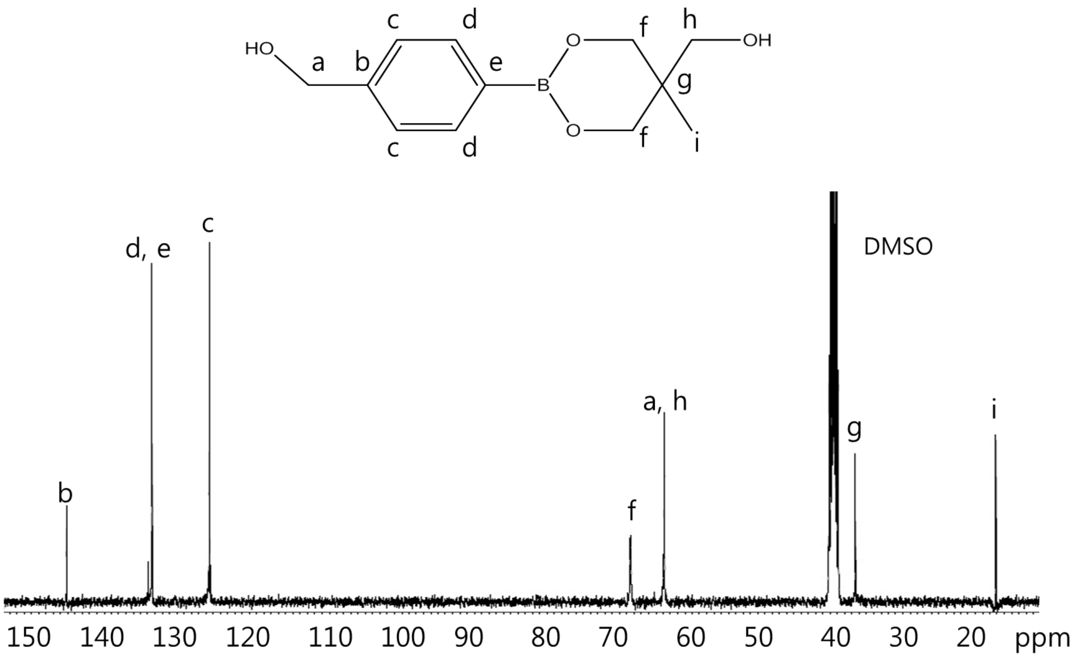

Figure S1. ^{13}C NMR spectrum of BRAP.

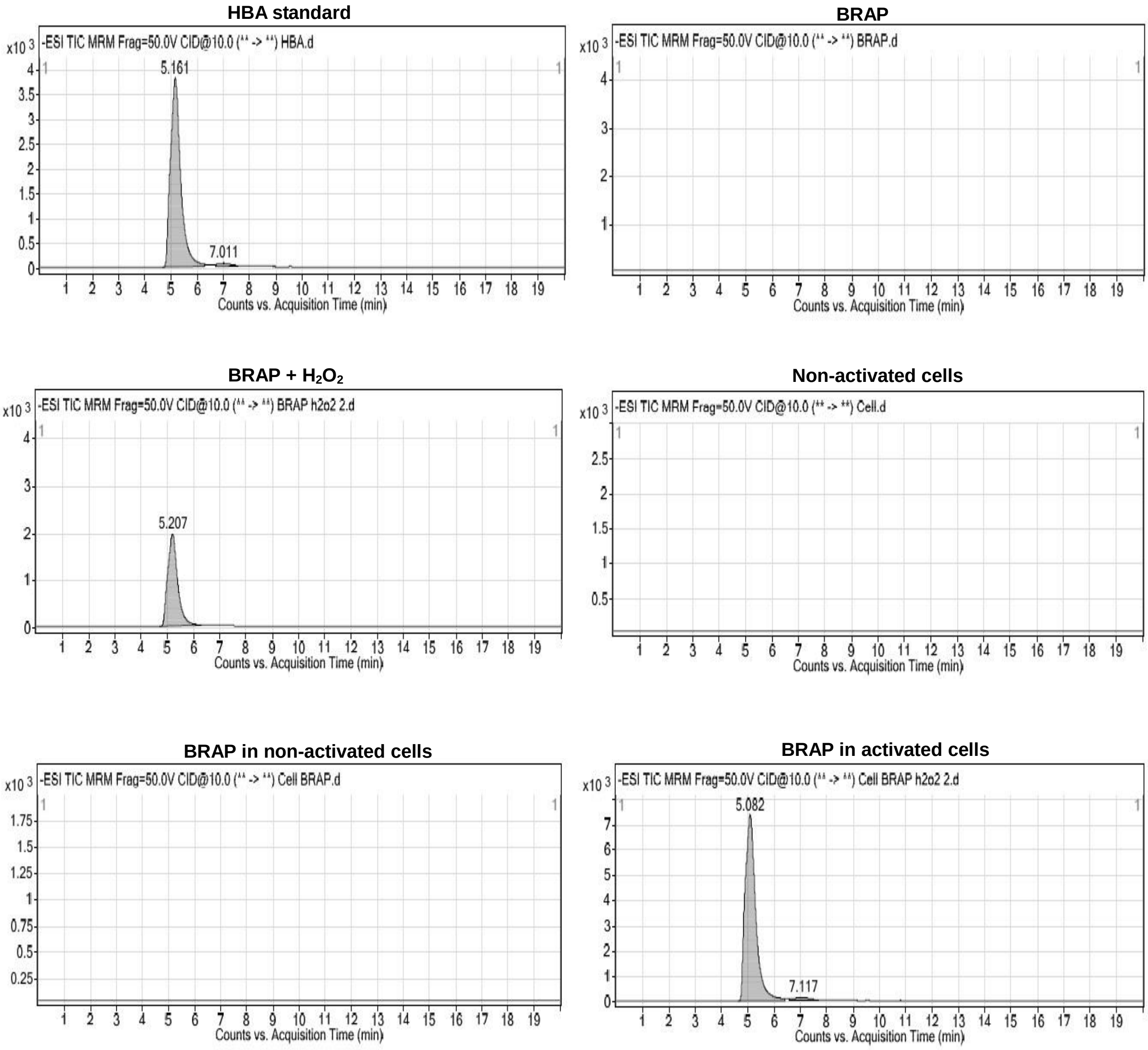

Figure S2. LC-MS/MS analysis of cell lyastes treated with BRAP.

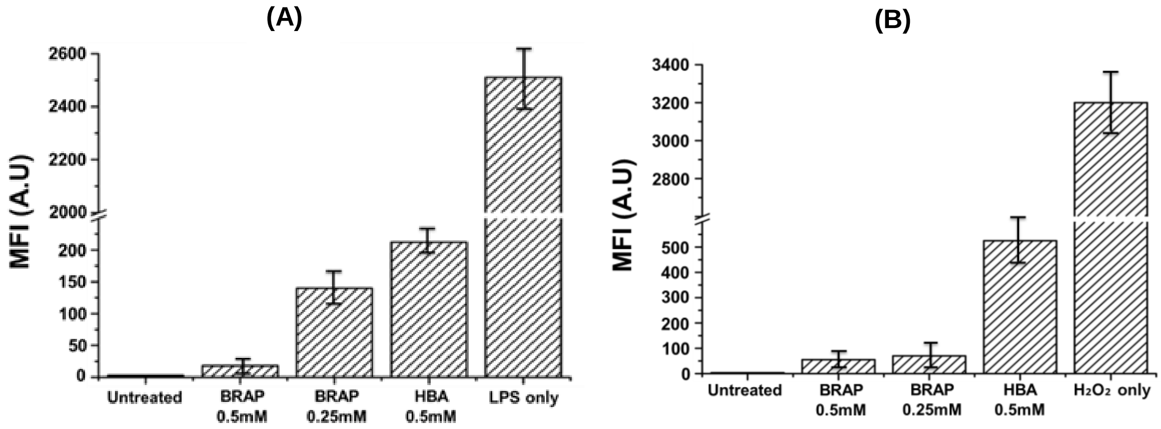

Figure S3. Quantification intracellular ROS generation in cells stimualted by LPS (A) and H_2O_2 (B). Valuse are mean ± s.d. (n=3).

(A)

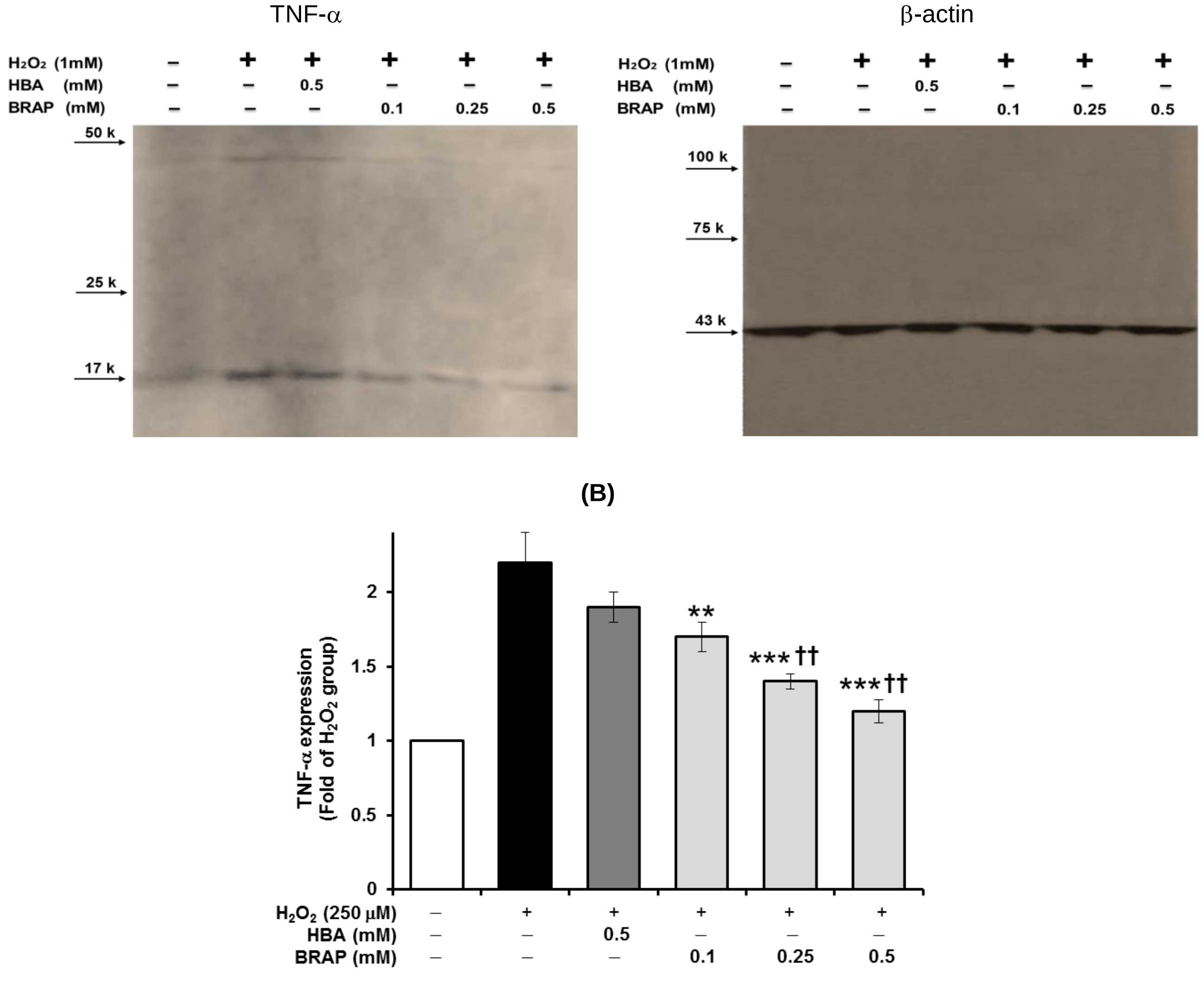

Figure S4. The effects of BRAP on the expression of TNF-α in RAW264.7 cells activated by H_2O_2. (A) A representative image of full lenght western blot images. (B) Quantification of the level of TNF-α in RAW264.7 cells. $^{**}p$<0.01, $^{***}p$<0.001 vs H_2O_2, $^{††}p$<0.01 vs HBA. Values are mean ± s.d. (n=3).

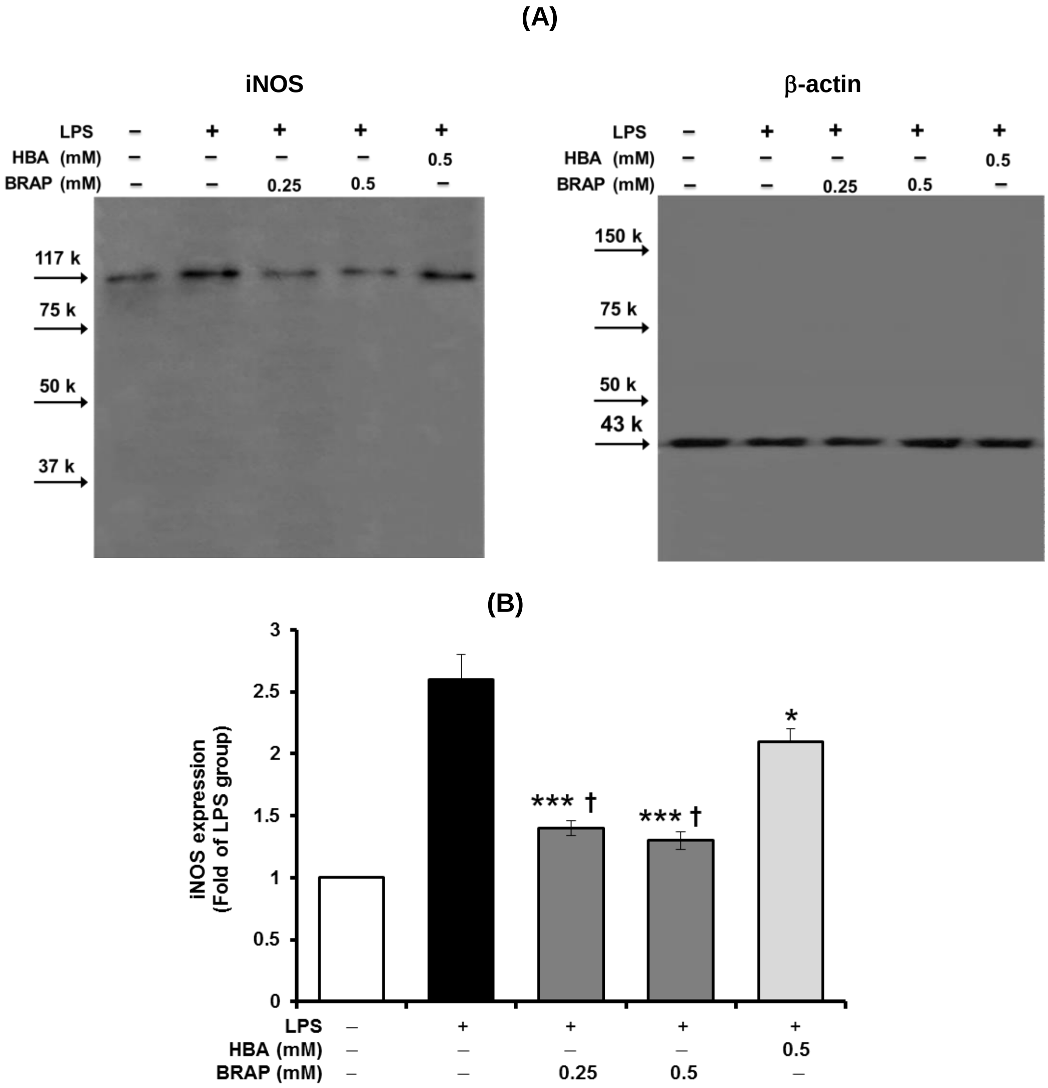

Figure S5. The effects of BRAP on the expression of iNOS in RAW264.7 cells activated by LPS. (A) A representative image of full lenght western blot images. (B) Quantification of the level of *i*NOS in RAW264.7 cells. ***p<0.001 vs LPS. †p<0.05 vs HBA. Values are mean ± s.d. (n=3).

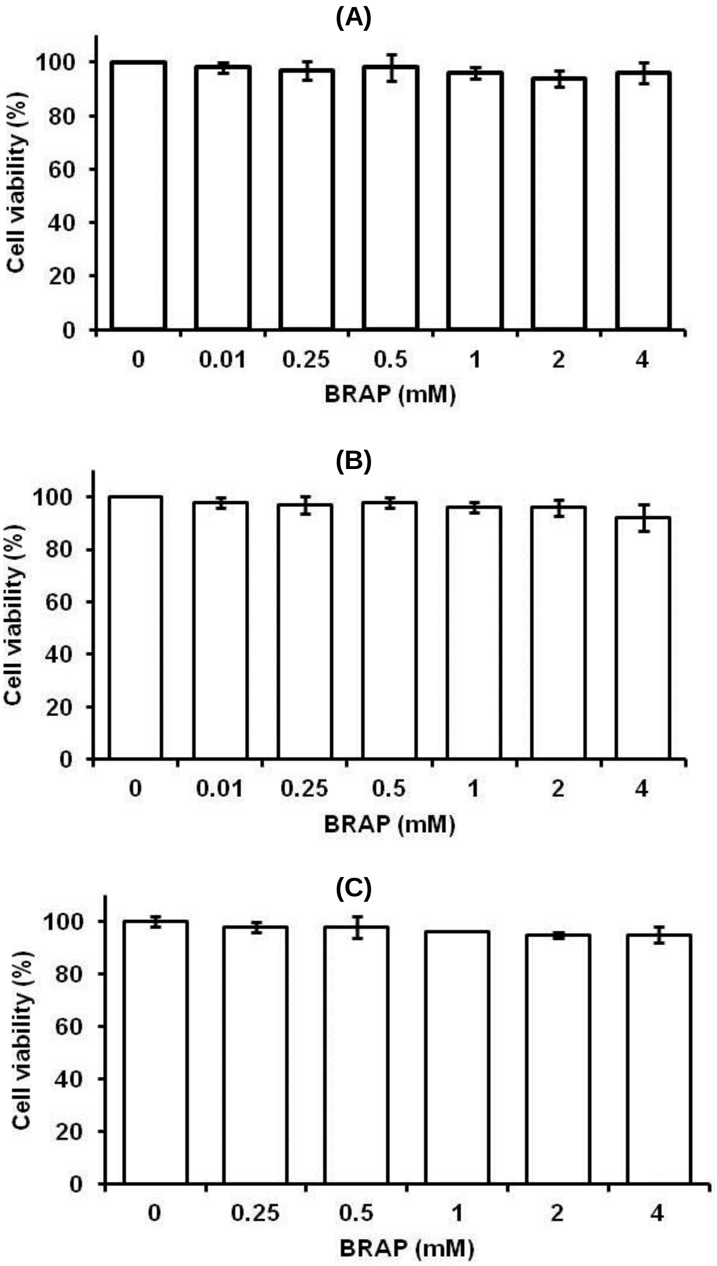

Figure S6. Biocompatibility profiles of BRAP assessed by MTT assay. (A) Mouse macrophages, (B) Mouse hepatocyte, (C) Adult rat ventricular cardiomyocytes. Values are mean ± s.d. (n=3).

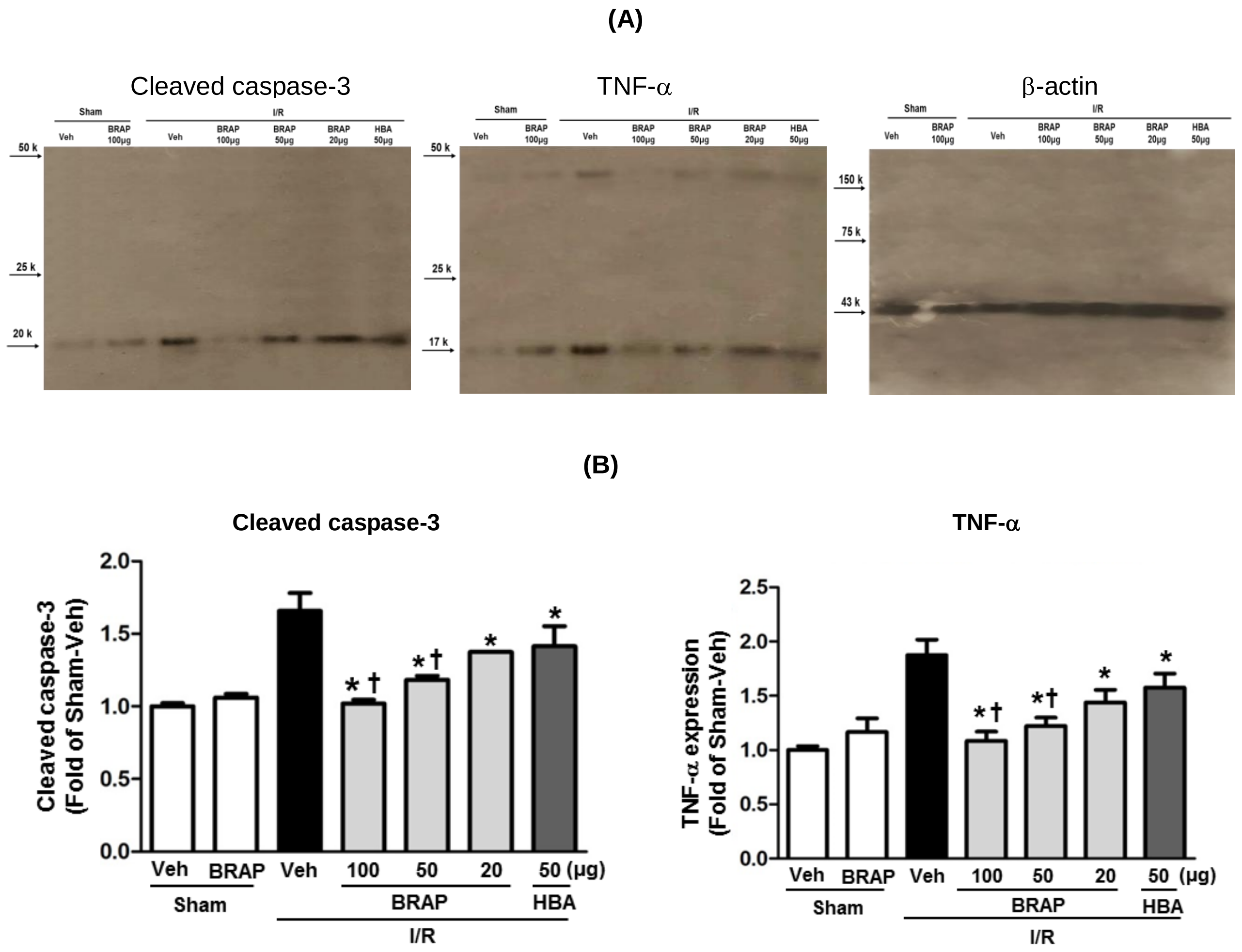

Figure S7. Anti-apoptotic activity and anti-inflammatory activities of BRAP in liver undergoing I/R. (A) A representative full-length western blot imaging of cleaved caspase-3 TNF-α and β-actin. (B) Quantification of the expression level of cleaved caspase-3 and TNF-α. *p<0.05 vs Veh, $^{\dagger}p$<0.05 vs HBA. Values are mean ± s.d. (n=3)..

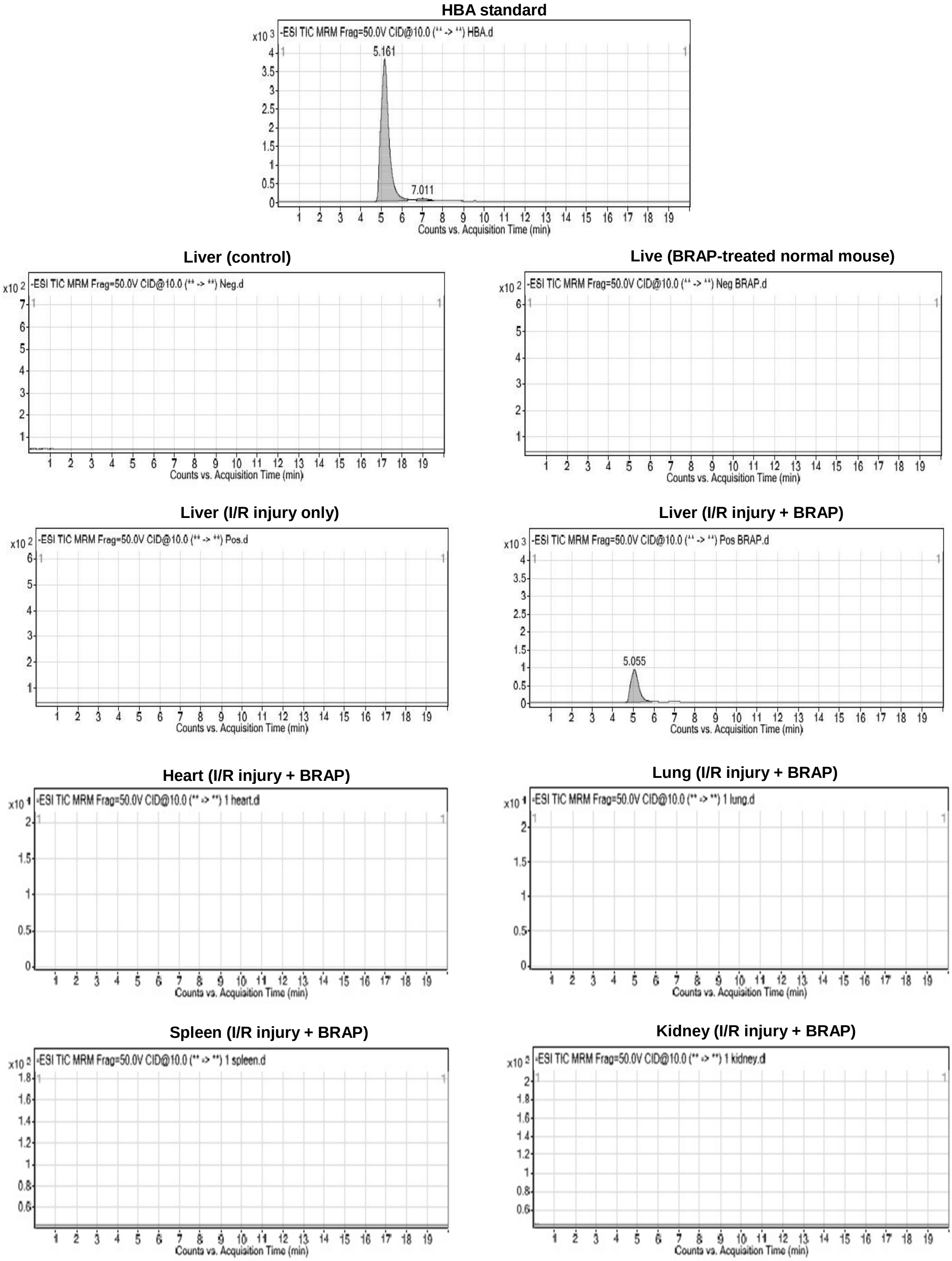

Figure S8. LC-MS/MS analysis of tissues of mice treated with BRAP. Reperfusion of hepatic artery was allowed for 4 h and the organs were removed for analysis.

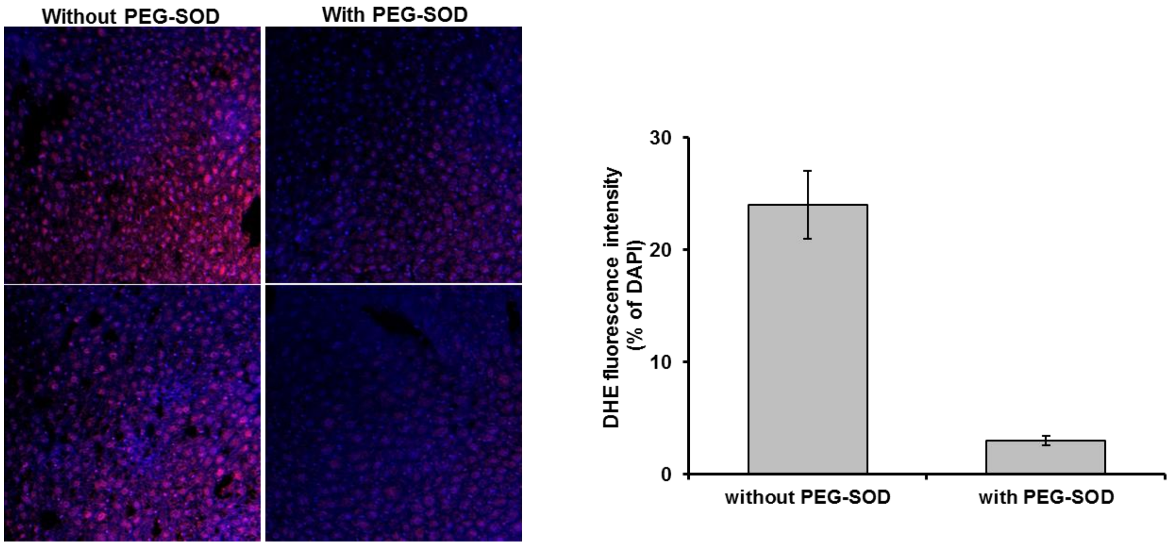

Figure S9. Representative images of DHE staining of liver tissues with and without PEG-SOD treatment. Prior to DHE staining, liver tissues exposed to I/R injury were pre-treated with PEG-SOD to verify that staining is specific for superoxide production. Values are mean ± s.d. (n=3).

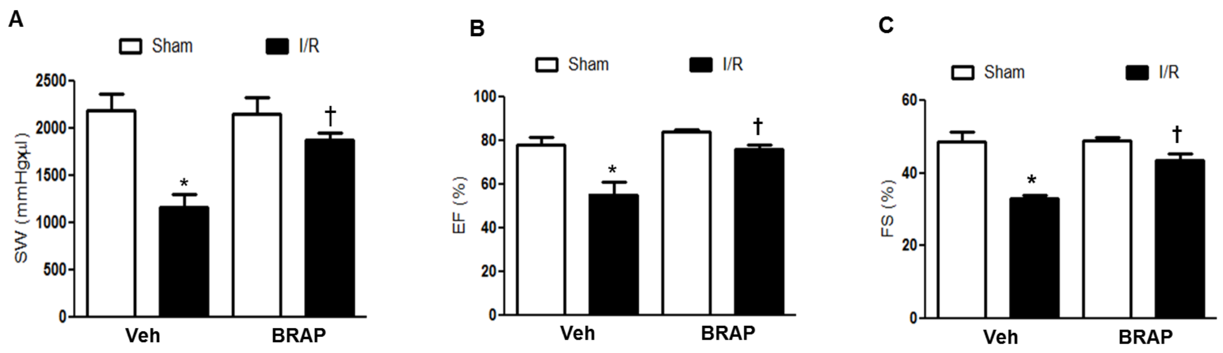

Figure S10. Stroke work (SW) (**A**), ejection fraction (EF) (**B**), and fractional shortening (FS) (**C**) with BRAP treatment after I/R injury. *p<0.05 vs. sham of each group, †p<0.05 vs Veh I/R. Values are mean ± s.d. (n=4-6/group).
